# Robot-assisted laparoscopic radical prostatectomy with early retrograde release of the neurovascular bundle and endopelvic fascia sparing

**DOI:** 10.1590/S1677-5538.IBJU.2015.0349

**Published:** 2017

**Authors:** George Augusto Monteiro Lins de Albuquerque, Giuliano Betoni Guglielmetti, Maurício Dener Cordeiro, William Carlos Nahas, Rafael Ferreira Coelho

**Affiliations:** 1 Instituto do Câncer de São Paulo, SP. Brasil;; 2 Divisão de Urologia, Faculdade de Medicina, Universidade de São Paulo, SP, Brasil

## Abstract

**Introduction:**

Robotic-assisted radical prostatectomy (RAP) is the dominant minimally invasive surgical treatment for patients with localized prostate cancer. The introduction of robotic assistance has the potential to improve surgical outcomes and reduce the steep learning curve associated with conventional laparoscopic radical prostatectomy. The purpose of this video is to demonstrate the early retrograde release of the neurovascular bundle without open the endopelvic fascia during RAP.

**Materials and Methods:**

A 51-year old male, presenting histological diagnosis of prostate adenocarcinoma, Gleason 6 (3+3), in 4 cores of 12, with an initial PSA=3.41ng/dl and the digital rectal examination demonstrating a prostate with hardened nodule in the right lobe of the prostate base (clinical stage T2a). Surgical treatment with the robot-assisted technique was offered as initial therapeutic option and the critical technical point was the early retrograde release of the neurovascular bundle with endopelvic fascia preservation, during radical prostatectomy.

**Results:**

The operative time was of 89 minutes, blood loss was 100ml. No drain was left in the peritoneal cavity. The patient was discharged within 24 hours. There were no intraoperative or immediate postoperative complications. The pathological evaluation revealed prostate adenocarcinoma, Gleason 6, with free surgical margins and seminal vesicles free of neoplastic involvement (pathologic stage T2a). At 3-month-follow-up, the patient lies with undetectable PSA, continent and potent.

**Conclusion:**

This is a feasible technique combining the benefits of retrograde release of the neurovascular bundle, the preservation of the pubo-prostatic collar and the preservation of the antero-lateral cavernous nerves.

ARTICLE INFO

**Available at: http://www.intbrazjurol.com.br/video-section/20150349_albuquerque_et_al/**

**Int Braz J urol. 2017; 43 (video #11): 782-782**

